# *Be a Mom*’s Efficacy in Enhancing Positive Mental Health among Postpartum Women Presenting Low Risk for Postpartum Depression: Results from a Pilot Randomized Trial

**DOI:** 10.3390/ijerph17134679

**Published:** 2020-06-29

**Authors:** Fabiana Monteiro, Marco Pereira, Maria Cristina Canavarro, Ana Fonseca

**Affiliations:** Center for Research in Neuropsychology and Cognitive Behavioral Intervention (CINEICC), Faculty of Psychology and Educational Sciences, the University of Coimbra Rua do Colégio Novo, 3000-315 Coimbra, Portugal; marcopereira@fpce.uc.pt (M.P.); mccanavarro@fpce.uc.pt (M.C.C.); ana.fonseca77@gmail.com (A.F.)

**Keywords:** web-based intervention, be a mom, randomized controlled trial, positive mental health, flourishing, postpartum period

## Abstract

In this study, we conducted a preliminary investigation of the efficacy of *Be a Mom*, a web-based self-guided intervention, in enhancing positive mental health among postpartum women at low risk for postpartum depression. Additionally, we examined *Be a Mom*’s efficacy regarding secondary outcomes as well as its acceptability and adherence. A total of 367 participants were randomly assigned to the *Be a Mom* group (*n* = 191) or to the waiting-list control group (*n* = 176) and completed baseline (T1) and postintervention (T2) assessments. The intervention group reported significant increases in positive mental health between T1 and T2 compared to the control group. Additionally, group effects were found for depressive and anxiety symptoms. A significantly higher proportion of participants in the *Be a Mom* group had an improvement trajectory (from not flourishing at T1 to flourishing at T2). A total of 62 (32.5%) women completed *Be a Mom*, and most would use it again if needed (*n* = 82/113; 72.6%). This study provides preliminary evidence of *Be a Mom*’s efficacy in increasing positive mental health among low-risk postpartum women. Our findings support mental health promotion strategies in the postpartum period and highlight the important role of web-based CBT interventions.

## 1. Introduction

The transition to motherhood is often depicted as a joyful period filled with excitement, but it also involves demanding adjustments to a new role and new responsibilities [1]. Research in this area has suggested that this is a period of increased mental health vulnerability due to the considerable number of stressors that are often experienced, such as increased physical health needs, adjusting to infant care tasks, changes in marital and social relationships, financial strains or transitioning back to work [2,3,4,5,6,7]. Even under optimal circumstances, the early postpartum period is full of challenging tasks. Although some women may present risk factors that make them more vulnerable to mental illness, such as postpartum depression (PPD; e.g., history of depression, low social support; [8]), this period constitutes a major life transition for all postpartum women, including low-risk women, who can also experience symptoms of depression and anxiety [9,10].

Mental illness during the postpartum period has increasingly been considered a high-priority public health problem due to the long-term negative implications for maternal health, infant health and development [11,12] and economic costs [13,14]. Even the presence of subclinical symptoms of depression has been associated with increased psychosocial difficulties (poorer maternal self-esteem, more negative and less negative positive affect) [15]. Several efforts have targeted the treatment and prevention of psychological disorders during this period, particularly PPD [16,17]. With respect to preventive interventions, there is evidence that cognitive behavioral therapy (CBT) is effective in preventing depression both among women who present risk factors for PPD (selective/indicated prevention) and among all women in the community (universal prevention), although the effects of these preventive interventions may be more robust among groups at a higher risk of perinatal depression [16,18].

In addition to prevention, the promotion of positive mental health has been increasingly recognized as a priority [19,20], with research suggesting the need to address positive mental health directly since interventions that are effective in reducing psychopathological symptoms are not necessarily effective in enhancing levels of positive mental health [21,22]. However, previous research has tended to consider women’s psychological adjustment to this period in terms of levels of depression and anxiety, and the promotion of positive mental health has been neglected in favor of a greater focus on the prevention and reduction of mental illness [23]. Positive mental health has received growing attention in recent years as research has shown that it is not simply the absence of mental illness [22,24]. Optimal levels of positive mental health involve the experience of high levels of emotional (positive feelings, e.g., feeling happy, satisfaction with life), psychological (optimal functioning in life, e.g., self-acceptance, purpose in life, personal growth) and social wellbeing (optimal social functioning, e.g., social integration and contribution) [24]. Keyes [24] conceptualized those with high levels of emotional, psychological and social wellbeing as flourishing individuals. Existing studies about the impact of flourishing underline the need for interventions that increase positive mental health. For instance, the presence of flourishing has been associated with better physical health and longevity [25,26], fewer health limitations of activities of daily living and fewer missed days of work [24] and may act as a buffer against future mental illness [27,28]. Although still rarely studied in the postpartum period, higher levels of positive mental health in mothers have been longitudinally associated with better development outcomes in children, specifically cognitive, communication and social development [29]. Thus, proactively addressing and assessing positive mental health in psychological interventions should be a complementary goal of psychological interventions in the postpartum period, including those targeting low-risk women.

Reaching all women in the postpartum period through traditional face-to-face interventions is difficult considering the high amount of human and economic costs involved [30]. Additionally, help-seeking rates for postpartum women with depressive symptoms range from 15% to 40% [31,32]. Suggested barriers that contribute to these low rates include feelings of shame, guilt and stigma and demands associated with infant care [33,34]. eHealth interventions have the potential to effectively address these barriers because they provide an opportunity to enhance the capacity and accessibility of mental health care and can be delivered at a very low cost and with privacy and convenience [35,36]. Moreover, these interventions have been shown to have long-term positive outcomes beyond the reduction of psychopathological symptoms, such as improvements in personal empowerment, self-esteem and quality of life [37]. In a recent review, the role of eHealth in the perinatal period was also highlighted as having the potential to be revolutionary if integrated into standard care [38].

In line with this, *Be a Mom* was developed to be a short-term, fully self-guided web-based intervention for Portuguese postpartum women. It was primarily developed to prevent PPD among at-risk women, and a previous pilot trial demonstrated its efficacy in reducing depressive and anxiety symptoms among a sample at high risk for PPD [39]. Although *Be a Mom* addresses the minimization of psychosocial risk factors for PPD (e.g., lack of social support, poor marital relationship), it does not focus solely on the minimization of such risk factors. Rather, *Be a Mom* specifically targets the development and strengthening of psychological competences and resources, namely, acceptance- and compassion-based skills. *Be a Mom* has already proven its efficacy in promoting self-compassion and emotion regulation skills among a high-risk sample for PPD [40]. Because the enhancement of psychological resources could be useful to all women in the postpartum period, *Be a Mom* may also be effective in the promotion of mental health among postpartum women presenting low risk for PPD. *Be a Mom* is grounded in CBT principles applied to the postpartum context and includes content based on the third wave of CBT, namely, acceptance- and compassion-focused approaches. Third-wave CBT approaches aim not only to reduce psychopathology but also to promote flourishing [41].

There is evidence of web-based CBT interventions as well as acceptance and compassion-based approaches enhancing positive mental health in general population samples [42,43]. However, there is still a lack of studies assessing the efficacy of these interventions in increasing positive mental health in the postpartum period. One study using an eHealth compassion-based intervention found that postpartum women in the intervention group showed an improvement in positive mental health compared to the control group [44], although the effects were small and only significant among participants with lower levels of positive mental health at baseline. Another study using an intervention based on positive psychology and metacognitive therapy only found a significant effect of the intervention with regard to emotional well-being [45]. The majority of web-based intervention studies in the perinatal period focus on the treatment or prevention of psychopathological symptoms using mostly high-risk samples [46]. However, given the characteristics of eHealth interventions and the importance of maternal mental health in influencing lifelong health outcomes, low-risk women may also benefit from these interventions.

Therefore, the present study reports the results of a pilot randomized controlled trial of the *Be a Mom* web-based intervention compared to a waiting-list control (WLC) group. The aims of this study were (1) to explore the efficacy of *Be a Mom* in enhancing positive mental health among low-risk postpartum women; (2) to investigate *Be a Mom*’s efficacy considering secondary outcomes (depressive and anxiety symptoms, maternal self-efficacy, empowerment and relationship satisfaction); and (3) to test *Be a Mom*’s acceptability, adherence and pattern of usage.

## 2. Materials and Methods 

### 2.1. Study Design 

This study was a two-arm, open-label, pilot randomized controlled trial on the efficacy of *Be a Mom* vs. a WLC for postpartum women presenting low risk for PPD. This study is part of a wider research project assessing *Be a Mom*’s efficacy as a web-based program for the promotion of maternal mental health during the postpartum period. The study was conducted in accordance with the Declaration of Helsinki, and the protocol was approved by the Ethics Committee of the Faculty of Psychology and Educational Sciences, University of Coimbra and was registered on ClinicalTrials.gov (NCT04055974). The extensions of the CONSORT 2010 checklist for pilot trials [47] and CONSORT-EHEALTH [48] were used for study reporting. 

### 2.2. Recruitment Procedure and Participants 

Participants were recruited online between January 2019 and February 2020. In addition to unpaid cross-posting, paid advertisements were placed on social media websites (Facebook and Instagram) targeting women aged 18–45 years old with interests in maternity topics. The tagline to advertise the study was “Did you have a baby in the last three months? We want to know if *Be a Mom* is effective in promoting postpartum women’s mental health, and you can help us! To know if you are eligible to participate in the study fill out the following form and we will contact you”. Participants who clicked on the link were then given information about the study’s goals and procedures, the participants’ and researchers’ roles, the voluntary nature of participation, and all aspects related to data protection (anonymity and confidentiality). Participants who gave online informed consent (by clicking the option “I understand and accept the conditions of the study”) answered a set of questions to assess eligibility criteria and provided their contact information (email address and telephone number). The inclusion criteria to participate in this study were a) being in the early postpartum period (up to 3 months postpartum); b) being 18 years or older; c) presenting low risk for PPD (having a score lower than 5.5 on the Postpartum Depression Predictors Inventory-Revised; [49]); d) having internet access at home; e) being a resident of Portugal; and f) understanding Portuguese. Exclusion criteria were the presence of a serious medical condition (physical or psychiatric) in the mother or in the infant (self-reported). Participants who did not meet eligibility criteria were sent an email informing them of the reason they could not participate in this study and advising them to seek professional help if needed. Because this study was part of a wider research project, women who presented risk factors for PPD were contacted by the research team to take part in a separate study to assess *Be a Mom*’s efficacy in preventing postpartum depressive symptoms (i.e., the primary outcome was depressive symptoms). 

A priori calculations indicated that a sample size of at least 200 participants at postintervention assessment was needed to assess preliminary evidence of efficacy for the primary outcome (detecting a small effect size [*d* = 0.10] with a statistical power of 0.80 in a two-tailed test, *p* < 0.05). Considering the dropout rate of approximately 35% in the pilot study of *Be a Mom* [39], at least 350 participants were needed for randomization. 

### 2.3. Randomization 

After completing the baseline assessment, eligible participants were randomly assigned (allocation ratio 1:1) to the intervention group (*Be a Mom*) or to the WLC group. Randomization was performed using a computerized random number generator. After randomization, participants received an email with information about their assigned group. The last author was responsible for randomization, and the first author was responsible for the enrollment and assignment of participants to either the *Be a Mom* group or the WLC group. 

### 2.4. Interventions

Participants in the intervention arm were invited to a password-protected website that contained the *Be a Mom* intervention (beamom.pt). Access to the program was free of cost, and no compensation was given to participants. *Be a Mom* has five sequential modules (Changes and Emotional Reactions; Cognitions; Values and Social Support; Couple’s Relationship (only presented to women in a relationship); PPD Alert Signs and Professional Help-seeking) and its contents are presented in an attractive format (simple text, animations, interactive exercises). The modules follow the structured and goal-oriented nature of CBT: first, the module’s goals are presented, followed by the thematic content and, finally, a homework activity at the end to guarantee continued therapeutic practice. Each module has an approximate length of 30–45 min, and women can interrupt it whenever they need to and resume when they are available. Asynchronous communication channels (e.g., reminders, email contact for program-related support) are available. The formative evaluation process that informed the design and the intervention components of *Be a Mom* is detailed elsewhere [50].

Participants were given the instructions that they should complete one module per week, but they were allowed to complete the program at their own pace. Those who did not register on *Be a Mom* were sent two email reminders during the course of the eight weeks given to complete *Be a Mom*. Participants who registered on the program and who had a valid telephone number were contacted via telephone by the first author approximately two weeks after registration. This contact aimed to clarify any questions regarding the flow of the program or help with difficulties accessing the website. Email reminders were sent automatically by the *Be a Mom* website to the participants if they went three, seven and 13 days without accessing it. Approximately two days after completing *Be a Mom*, participants were sent an email with the postintervention assessment. Those who did not complete the program were sent an email with the postintervention assessment eight weeks after randomization. 

Participants in the WLC arm were offered no intervention but were informed that they would receive access to *Be a Mom* at the end of the study. They were asked to complete the postintervention assessment protocol at the same time as the participants in the *Be a Mom* group (eight weeks after randomization). All participants could access usual care from their health services.

Outcome variables were assessed at baseline (Time 1—T1) and eight weeks after randomization (Time 2—T2) by self-report using the survey platform Limesurvey^®^. To reduce attrition, email and text message reminders were sent on an alternate basis each week for one month to women in both groups who failed to complete T1 and T2 online questionnaires.

### 2.5. Measures 

Women’s sociodemographic (e.g., age, marital status, number of children, employment status, educational level, household monthly income, and residence), clinical (psychopathology history) and infant-related data (e.g., infant’s age, infant’s sex and infant’s gestational weeks at birth) were collected through a self-report questionnaire developed by the researchers.

To identify women presenting low risk for PPD, the Portuguese version of the Postpartum Depression Predictors Inventory-Revised (PDPI-R) was used [51]. This questionnaire comprises 39 items answered on a dichotomous scale (yes vs. no, except for the first two items in which participants report their marital and socioeconomic status). The PDPI-R total score ranges from 0 to 39. Higher scores indicate increased risk for PPD. In Portuguese validation studies, a score of 5 or lower is indicative of lower PPD risk [49].

#### 2.5.1. Primary Outcome-Positive Mental Health

Positive mental health was assessed using the Mental Health Continuum Short Form (MHC-SF; [52]; Portuguese Version [PV]: [53]). The MHC-SF comprises 14 items divided into three dimensions: emotional (3 items; e.g., “During the past month, how often did you feel happy?”), social (five items; e.g., “During the past month, how often did you feel that you had something important to contribute to society?”) and psychological wellbeing (six items; e.g., “During the past month, how often did you feel that your life has a sense of direction or meaning to it?”). Each item is rated on a six-point response scale from 0 (*never*) to 5 (*every day*) in reference to the last month. In Portuguese psychometric studies, only the use of the total score was recommended as no adequate support was found for the use of the subscales as measures of distinct dimensions. The MHC-SF can be scored continuously (scores range from 0 to 70, and higher scores indicate better positive mental health) or categorically considering mental health status (flourishing, moderate mental health, languishing). Using Keyes’ criteria [52], women who answered *every day* or *almost every day* at least once in the emotional wellbeing subscale and at least six times in the psychological and social wellbeing subscales were categorized as flourishing. Women who answered *never* or *once or twice* for at least one item in the emotional wellbeing subscale and at least six items in the psychological and social wellbeing subscales were categorized as languishing. Finally, women who did not fit the criteria for either flourishing or languishing were considered moderately mentally healthy. In this study, we categorized participants as flourishing and not flourishing (including both languishers and those with moderate mental health). The Cronbach’s alpha values in this study ranged from 0.90 (intervention group—T1) to 0.93 (intervention group—T2).

#### 2.5.2. Secondary Outcomes 

The Edinburgh Postnatal Depression Scale (EPDS; [54]; PV: [54]) was used to assess postpartum depressive symptoms. EPDS comprises 10 items (e.g., “I have blamed myself unnecessarily when things went wrong”) that are rated with an individualized four-point response scale (ranging from 0 to 3). The total score ranges between 0 and 30, and higher scores are indicative of more severe depressive symptoms. In this study, the Cronbach’s alpha values ranged from 0.80 (intervention group—T1) to 0.86 (control group—T2).

The Anxiety Subscale of the Hospital Anxiety and Depression Scale (HADS-A; [55]; PV: [56]) was used to assess anxiety symptoms. This widely used seven-item subscale (e.g., “Worrying thoughts go through my mind”) employs a four-point response scale (ranging from 0 to 3) that assesses the presence of anxiety symptoms in the week prior to completion. Higher scores denote higher anxiety symptoms. In this study, the Cronbach’s alpha values ranged from 0.76 (intervention group—T1) to 0.82 (intervention group—T2).

The Empowerment Scale (ES; [57]; PV: [58]) was used to assess empowerment. The ES is a self-reported questionnaire with 20 items (e.g., “I am usually confident about the decision I make”) that are rated with a four-point scale ranging from 1 (*strongly agree*) to 4 (*strongly disagree*). Higher scores indicate higher levels of empowerment. The Cronbach’s alpha values in this study ranged from 0.79 (control group—T2) to 0.89 (intervention group—T2).

Women’s perception of self-efficacy in the mothering role was assessed using the Perceived Maternal Parenting Self-Efficacy (PMP S-E; [59]). This measure comprises 20 items (e.g., “I am good at understanding what my baby wants”) rated with a four-point scale ranging from 1 (*strongly disagree*) to 4 (*strongly agree*). Higher scores indicate higher levels of perceived self-efficacy. In this study, the Cronbach’s alpha values ranged from 0.91 (control group—T2) to 0.95 (intervention group—T2).

The Satisfaction subscale of the Investment Model Scale (IMS-S; [60]; PV: [61]) was used to assess satisfaction in the relationship with the woman’s partner. This subscale comprises five items (e.g., “Our relationship makes me very happy”) answered on nine-point scale ranging from 0 (*do not agree at all*) to 8 (*completely agree*). Higher scores indicate higher satisfaction with the relationship. In this study, the Cronbach’s alpha values ranged from 0.81 (intervention group—T1) to 0.83 (control group—T2).

#### 2.5.3. Be a Mom’s Web System Data

Data were collected through the *Be a Mom* website regarding the number of completed modules and pages accessed in each module, number of logins, average minutes spent on the website at each login, number of finished exercises and number of times each audio exercise was played. 

#### 2.5.4. Be a Mom’s Acceptability and Experience

At the postintervention assessment, the intervention group completed an additional set of questions referring to *Be a Mom*’s acceptability. Participants were asked to answer questions with a two-point response scale (1 = *not applicable to me*; 2 = *applicable to me*) regarding their satisfaction with the help provided by the program, their intentions to use it again if needed and to recommend it to a friend, usefulness/relevance of the information learned and demandingness. Additional questions were presented about the participant’s experience using *Be a Mom*; these were open-ended questions regarding the presence of others when accessing the program and reasons for not completing all modules of *Be a Mom*, when applicable. 

### 2.6. Data Analysis

Statistical analyses were conducted in accordance with the intention-to-treat principles following the CONSORT statement [62] so that all participants who completed baseline assessment were included even if they did not complete postintervention assessment. Data were analyzed using the Statistical Package for Social Sciences (IBM SPSS, version 23.0 (IBM Corp., Armonk, NY, USA)). Descriptive statistics and comparison tests (t-tests and chi-squared tests) were computed for sample characterization and to examine *Be a Mom*’s usage and acceptability. Comparison analyses were also conducted between completers and dropouts and completers and non-completers. Dropout was defined as not completing the postintervention assessment regardless of the number of modules completed, and non-completers were defined as not completing the intervention. 

Linear mixed models (LMMs) were used to determine the effects of the intervention over time on primary and secondary outcomes. LMMs are particularly helpful in longitudinal studies with missing data because they allow incomplete cases to be included in the analysis. All available data are used to obtain parameter estimates with small bias in the presence of data missing completely at random or missing at random [63]. Group, time and time by group interaction and covariates (variables presenting statistically significant differences between intervention and control groups at T1 and between completers and dropouts at T2: previous history of psychopathology, infant’s age and category of positive mental health) were fitted as fixed effects. Participants were included as a random intercept. An LMM with an autoregressive covariance matrix was conducted for each outcome with the assumption of data missing completely at random (Little’s MCAR test *χ^2^* = 415.21, *p* = 0.938). Missing endpoints at posttest ranged from 119/367 (32.4%) on the MHC-SF to 127/367 (34.6%) on the IMS-S. 

Additionally, chi-squared tests were used to examine differences in the proportion of patterns of change as a function of group (intervention vs. control group) considering the primary outcome. Based on the cutoff scores of the MHC-SF, participants were categorized as flourishing or not flourishing at both T1 and T2. Participants were then classified in accordance with their pattern of change from T1 to T2: (a) maintenance—flourishing (if they were flourishing at both T1 and T2); (b) maintenance—not flourishing (if they were not flourishing at both T1 and T2); (c) deterioration (if they were flourishing at T1 and not flourishing at T2); and (d) improvement (if they were not flourishing at T1 and flourishing at T2).

## 3. Results

### 3.1. Participant Characteristics

**Figure 1:**
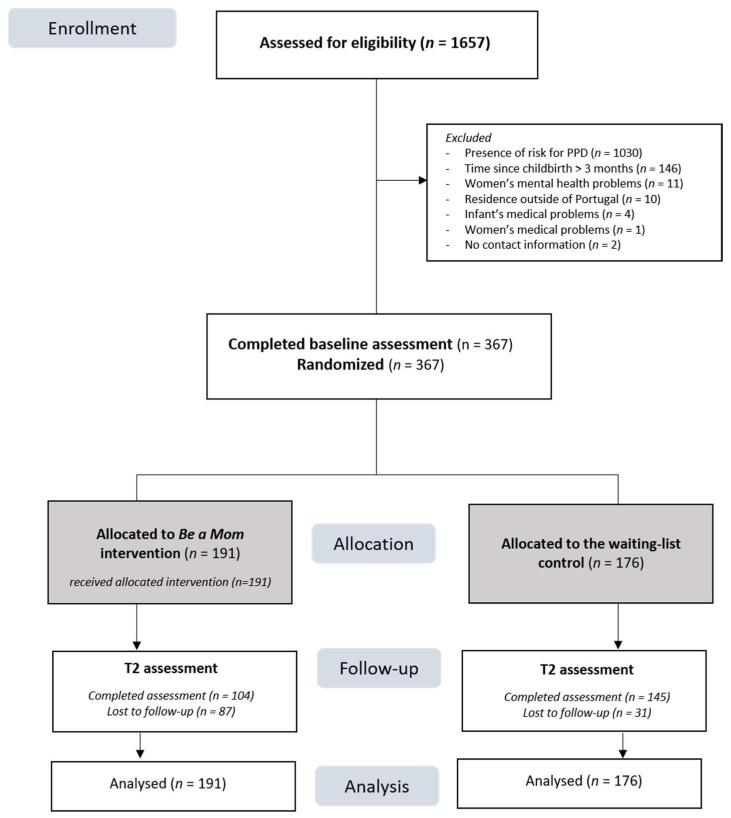
Flowchart of the participants in the study.

Figure 1 shows the flow diagram of the participants throughout the study period. Of the 1657 women who were screened for eligibility, 72.5% (*n* = 1202) were excluded due to not meeting eligibility criteria (mostly because they presented risk for PPD; *n* = 1030, 85.7%). Of the 455 eligible participants, 367 completed the baseline assessment and were randomized and allocated to the intervention group (*n* = 191) or to the WLC group (*n* = 176).

Table 1 summarizes the baseline sociodemographic and clinical characteristics of the intervention and control groups. There were no significant differences in most sociodemographic and clinical characteristics. However, a significantly higher proportion of participants in the intervention group had a previous history of psychopathology than in the control group (25.1% vs. 14.2%, *χ^2^* = 6.86, *p* = 0.009). The control group also had a significantly higher proportion of participants who were flourishing (67% vs. 57.1%, *χ^2^* = 3.86, *p* = 0.049).

The overall retention rate at the postintervention assessment was 67.8%. The intervention arm had significantly higher loss to follow-up than the control arm (intervention group: *n* = 87, 45.5% vs. control group: *n* = 31, 17.6%, *χ^2^* = 32.77, *p* < 0.001). Potential differences between completers and dropouts on baseline sociodemographic and clinical characteristics were explored but did not reveal any significant differences. The only exception was infant age, with infants of the participants who dropped out of the study being older than the infants of those who completed the postintervention assessment (*M* = 2.11 months, *SD* = 0.97 vs. *M* = 1.77 months, *SD* = 1.20, *t* = −2.68, *p* = 0.008). The proportion of women who had psychological/psychiatric treatment after the baseline assessment was also similar in both groups (intervention group: *n* = 8, 7.7% vs. control group: *n* = 7, 4.9%, *χ^2^* = 0.85, *p* = 0.356).

### 3.2. Be a Mom’s Preliminary Evidence of Efficacy: Comparison with the Control Group

Table 2 presents the estimated marginal means of all outcome measures and fixed effects for time, group and time × group interaction as well as for covariates (psychopathology history, infant’s age and category of positive mental health at baseline). 

**Figure 2:**
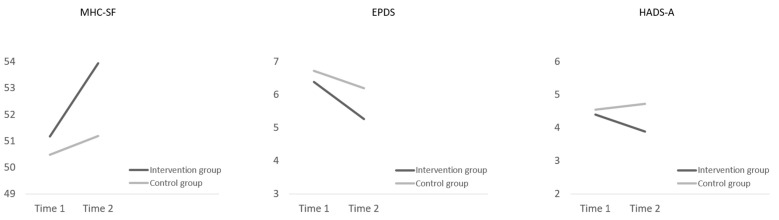
Intervention and control group trajectories for the Mental Health Continuum-Short Form, the Edinburgh Postnatal Depression Scale and the Anxiety subscale of the Hospital Anxiety and Depression Scale from T1 to T2 (based on mean estimates from linear mixed models).

Regarding the primary outcome, the LMM revealed a significant effect of time × group interaction, with women in the intervention group reporting a greater increase in positive mental health levels than participants in the control group (see Figure 2). 

Regarding the secondary outcomes, significant effects of group were found for depressive and anxiety symptoms. Specifically, we found that these symptoms were higher overall in the control group. Although no significant time × group interactions were found, Figure 2 shows that a greater decrease in depressive and anxiety symptoms was found from T1 to T2 in the intervention group. 

Regarding the remaining secondary outcomes, no interaction effects of time × group were found. For maternal self-efficacy and relationship satisfaction, significant effects of time were found. In both the intervention and control groups, there was a decrease in satisfaction in the relationship with the partner and an increase in maternal self-efficacy from T1 to T2, but there were no significant differences between the *Be a Mom* group and the WLC group. 

**Figure 3:**
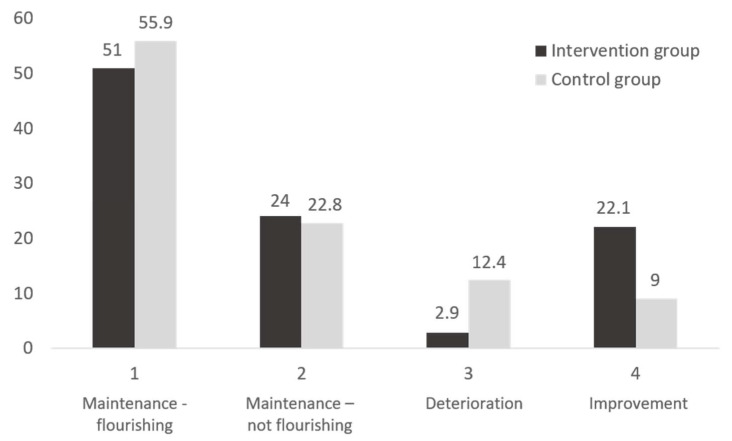
Trajectory of participants in the intervention and control groups from Time 1 to Time 2 regarding category of positive mental health. Maintenance—flourishing: flourishing at both T1 and T2; Maintenance—not flourishing: not flourishing at both T1 and T2; Deterioration: flourishing at T1 and not flourishing at T2; Improvement: not flourishing at T1 and flourishing at T2.

Figure 3 shows that a significantly higher proportion of women in the intervention group had an improvement trajectory (from not flourishing at T1 to flourishing at T2) compared to women in the control group (*n* = 21, 20.2% vs. *n* = 14, 9.7%, *χ^2^* = 10.59, *p* = 0.014). Additionally, a higher proportion of women in the control group had a deterioration trajectory (from flourishing at T1 to not flourishing at T2) compared to women in the intervention group (*n* = 18, 12.4% vs. *n* = 4, 3.8%).

### 3.3. Adherence to the Intervention, Be a Mom’s Usage and Acceptability 

A total of 191 participants received an email invite to access the *Be a Mom* website. Of these, 62 (32.5%) completed the intervention, 23 (12%) completed half of the program, and 20 (10.5%) did not register on the website and initiate the intervention. Of the participants who registered on the *Be a Mom* website, 29 (17%) participants did not complete any module. 

Considering all 171 participants who registered on the *Be a Mom* website, the average number of logins was 6 (*SD* = 3.78, range: 1–20) and the average number of minutes spent on the website in each login was 16 (*SD* = 11.80, range: 0–73). The majority of participants finished all four exercises of module 1 (*n* = 128, 74.9%) and the two exercises of module 2 (*n* = 103; 60.2%). Considering the participants who completed modules three, four and five, most of them finished all exercises proposed (four in module three, two in modules four and five; *n* = 73/80, 91.3%; *n* = 58/60, 96.7%; *n* = 30/59, 50.8%, respectively). Most of the participants who completed module 2 listened to the two audio exercises provided (observing thoughts and distancing of thoughts) (*n* = 78/103, 75.7% and *n* = 48/103, 46.6%, respectively). 

Moreover, 113 participants in the intervention group answered a questionnaire about their experience using *Be a Mom* and its acceptability. Of these, 55 (48.7%) did not complete the intervention. Regarding the reasons for not completing *Be a Mom*, 53 (96.4%) participants highlighted lack of time, one (1.8%) participant answered that it was due to personal issues and one (1.8%) answered that *Be a Mom* was not useful in her case. Most women accessed *Be a Mom* on their own (*n* = 102; 90.3%). The remaining 11 participants accessed *Be a Mom* with their partners (*n* = 10; 90.9%) and other family members (*n* = 1; 9.1%), and all considered it beneficial for them and for the other person.

Of the 113 participants who answered the acceptability questionnaire, 65.5% (*n* = 74) were satisfied with the help provided by *Be a Mom*, 85% (*n* = 96) would recommend it to a friend, and 72.6% (*n* = 82) would use it again if needed. Moreover, 92% (*n* = 104) of participants rated the quality of *Be a Mom* as good/excellent. Additionally, 74.3% (*n* = 84) felt that they had learned relevant information with *Be a Mom*. Finally, 21 women (5.7%) considered participating in *Be a Mom* to be too demanding.

When comparing completers and non-completers of *Be a Mom*, a higher proportion of completers was satisfied with the help provided by *Be a Mom* (*n* = 43, 74.1% vs. *n* = 31, 56.4%, *χ^2^* = 3.95, *p* = 0.047). Moreover, when compared to non-completers, a higher proportion of completers would recommend *Be a Mom* to a friend (*n* = 54, 93.1% vs. *n* = 42, 76.4%, *χ^2^* = 6.19, *p* = 0.013), would use it again if they needed (*n* = 48, 82.8% vs. *n* = 34, 61.8%, *χ^2^* = 6.22, *p* =.013) and felt that they learned relevant information with the program (*n* = 49, 84.5% vs. *n* = 35, 63.6%, *χ^2^* = 6.43, *p* = 0.011). A higher proportion of non-completers considered participating in the *Be a Mom* program to be too demanding compared to completers (*n* = 17, 30.9% vs. *n* = 4, 6.9%, *χ^2^* = 10.76, *p* = 0.001). 

## 4. Discussion

The current study examined the efficacy of a web-based intervention, *Be a Mom*, in increasing positive mental health among postpartum women presenting low risk for PPD compared to a waiting-list condition. The efficacy of *Be a Mom* on secondary outcomes (depressive and anxiety symptoms, maternal self-efficacy, empowerment and relationship satisfaction) was also examined. The results of our study suggest that *Be a Mom* was superior to the WLC in increasing positive mental health. Additionally, there was a decreasing trend over time in depressive and anxiety symptoms in the *Be a Mom* group. *Be a Mom* was also shown to be an acceptable web-based intervention among low-risk postpartum women with satisfactory adherence and usage. 

The significant improvement of positive mental health in the *Be a Mom* group is in line with previous evidence supporting the efficacy of web-based CBT interventions to promote positive mental health [42,43]. However, this is one of the first trials to successfully demonstrate this in the postpartum period. Additionally, our results showed that *Be a Mom* increased the proportion of flourishers over time. Conversely, a higher proportion of women in the control group had a deterioration trajectory (from flourishing at baseline to not flourishing at postintervention assessment). This is an important finding given the demonstrated importance of flourishing in protecting against future adversities and mental disorders, such as anxiety and depression [27,28], as well as its association with several health and psychosocial outcomes [24,64]. Thus, *Be a Mom* could have an important impact on mental health outcomes in the long term and, as such, enhance public mental health. Future studies using long-term assessments could test this hypothesis.

Regarding the secondary outcomes, the interaction effects of time and group failed to reach statistical significance. However, regarding depressive and anxiety symptoms, we found significant group effects (these symptoms were higher in the control group) as well as a trend of a greater decrease in the intervention group, particularly for anxiety symptoms. When looking at mean estimates, we found that from T1 to T2, there was an increase in anxiety symptoms in the control group contrasting with a reduction in the intervention group. This suggests that participants who received the *Be a Mom* program had greater benefits than those in the control group. However, although the efficacy of *Be a Mom* in reducing depressive and anxiety symptoms was previously demonstrated [39], it is important to note that *Be a Mom* seems to not have a substantial impact on such symptoms among low-risk postpartum women. A possible explanation may be the relatively low levels of depressive and anxiety symptoms at baseline, which leaves limited space for improvement. 

Moreover, we found an increase over time in maternal self-efficacy in both study groups. This is consistent with the existing literature [65,66]: infants’ demands become more predictable as they grow older, providing the opportunity to increase the mother’s ability to successfully perform childcare tasks. However, we found that *Be a Mom* did not have a significant impact on the maternal self-efficacy of low-risk women. Although *Be a Mom* does not target caregiving behaviors, we expected that the emotional state of participants would influence the assessment of their self-efficacy, in line with Bandura’s self-efficacy theory [67] and previous studies associating depressive symptoms with lower maternal self-efficacy [68]. This result is consistent with those previously found on the efficacy of *Be a Mom* among a high-risk sample [39] and provides further evidence that *Be a Mom* may not be a suitable intervention to contribute to maternal confidence and self-efficacy, at least in the short term.

With respect to the results on empowerment, small, nonsignificant differences were found between the groups over time. Looking at mean estimates, the results suggest an increase in empowerment in the *Be a Mom* group compared with a decrease in the control group. A previous trial in the general population highlighted the significant impact of a web-based intervention on empowerment levels [37], suggesting that individuals who access the intervention may feel that they are more informed and involved in managing their own health outcomes. However, this was not found for *Be a Mom*. Future trials with follow-up assessments could provide more information on the pattern of this finding. 

Finally, we found that both groups presented a decline in relationship satisfaction over time. Previous studies have emphasized that the arrival of an infant can strain the romantic relationship and produce changes in the couple, with a decline in satisfaction [69]. Although *Be a Mom* has a module that focuses on the changes that can happen within the couple during this period, most participants (90.3%) accessed *Be a Mom* on their own. The strategies that are presented (e.g., acknowledging the difficulties experienced by the other member of the couple, assertive and open communication skills, accepting differences in backgrounds and parental values) should ideally be implemented in a joint effort by the two members of the couple. In the future, further efforts and instructions to involve partners when accessing *Be a Mom* could help clarify its impact on relationship satisfaction.

In addition to the results on the efficacy of *Be a Mom*, our study provided results on *Be a Mom*’s acceptability as well as its usage and adherence. Extending on previous acceptability results among postpartum women presenting risk factors for PPD [39], *Be a Mom* appears to also be an acceptable option for low-risk postpartum women. However, adherence to the intervention was relatively low (only 32.5% women completed *Be a Mom*). This result is congruent with those found by previous research with web-based interventions applied to universal samples during the perinatal period [44,45] and could be explained by the hectic and challenging period women are experiencing, which leaves them with limited time for themselves. Additionally, *Be a Mom* is a fully self-guided intervention that was delivered to a low-risk sample with relatively good levels of overall mental health at baseline. It is possible that participants perceived that they did not need to complete the intervention and prioritized their limited time with other tasks. Nevertheless, those who completed the intervention or accessed half of the modules adhered to most of the exercises that were proposed. 

Overall, the results of our study are very encouraging: they support mental health promotion strategies in the postpartum period and highlight the important role of web-based CBT interventions in achieving this. First, the postpartum represents an appropriate period to implement mental health promotion strategies given the pervasive consequences of mental health problems during this period and how they might shape children’s development and health outcomes in the long term [11]. Second, the finding that low-risk women may significantly benefit from *Be a Mom* gives strength to the argument that mental health promotion strategies at a population level are needed. Growing research has emphasized that it is not sufficient to target only groups suffering from mental disorders or at-risk groups as new cases of mental illness will emerge [70,71]. A small change in the average level of positive mental health per individual could lead to significant benefits in population terms and shift the population distribution of positive mental health [70], with a possible impact on the prevalence and burden of mental disorders. Finally, *Be a Mom*’s brief and unguided format provides the opportunity to be delivered with low costs and to be easily disseminated at a population level. If implemented in healthcare, *Be a Mom* could be used as an early intervention of a stepped-care model, which is highly recommended by international guidelines [72], and could consequently provide a more efficient use of resources. 

Although the results of our study are innovative and may add important input to public health policies, some limitations should be considered when interpreting our findings. First, the generalizability of the results is limited because the participants in the current study were self-selected, and it is possible that women with an interest in the topic were more likely to participate. Moreover, one criterion to be included in this study was having internet access and this could also represent a selection bias. Additionally, our sample was mainly composed of highly educated and employed women. Future studies could build on current findings by investigating the efficacy of *Be a Mom* in more heterogeneous and representative samples. Second, there was a high attrition rate and low adherence in the intervention group. This is in line with previous intervention studies in the perinatal period [46] and, as previously mentioned, could be explained by the demanding nature of the early postpartum period. The lower attrition rate in the control group could be explained by the added incentive of gaining access to *Be a Mom* by continuing participation in the study. Future intervention studies during the postpartum period need to take into account the challenging and time-restricted period women are experiencing when designing research. Brief assessment protocols could help improve attrition rates. Additionally, future studies using the *Be a Mom* program must be mindful of this study’s low adherence. Although a great proportion of participants answered that they did not complete the program due to lack of time, it is important to understand the reasons underlying this low rate and how much does it reflect the demandingness of the early postpartum period or the intervention’s structure, length and contents. Third, the lack of an active control group does not allow us to rule out the possibility that the effects found were due to social desirability or placebo effects. Fourth, while the present study found evidence of the efficacy of *Be a Mom* in enhancing positive mental health, the observed effect was only a short-term improvement. Thus, the results presented are best interpreted as providing promising evidence. Future RCTs with follow-up assessments are needed to test *Be a Mom*’s efficacy in producing enduring positive effects. Examining the long-term impact of this approach with added input from a cost-effectiveness analysis could meaningfully inform public health policies. Finally, the mechanisms that explain the participants’ response to treatment were not directly explored in this study. Because *Be a Mom* was developed to mainly target psychological resources such as self-compassion and emotion regulation, further studies exploring whether these mechanisms are involved in explaining the increase in levels of positive mental health are needed. 

## 5. Conclusions

The promotion of positive mental health in the perinatal period has received very little attention to date. This is one of the first trials to test a web-based CBT intervention for this purpose among postpartum women presenting low risk for PPD. Given our findings, *Be a Mom* could be considered a new mental health promotion strategy among Portuguese postpartum women. The results of this pilot trial support its preliminary efficacy in increasing positive mental health to a flourishing mental health status with the potential to reduce depressive and anxiety symptoms among a nonclinical population. As an unguided web-based intervention, *Be a Mom* offers an accessible intervention option that could easily be disseminated among all postpartum women. Further research with a larger sample and long-term follow-up assessments is required to consolidate our findings and significantly inform public health policies. 

## Figures and Tables

**Table 1:** Participants’ sociodemographic and clinical characteristics at baseline.

Variables	Intervention Group (*n* = 191)	Control Group (*n* = 176)	*t*/χ^2^
	*M* (*SD*)/*n* (%)	*M* (*SD*)/*n* (%)	
Age	33 (4.04)	33 (4.43)	−0.14
Marital status			0.53
Married/co-habiting	183 (95.8)	170 (96.6)	
Single	4 (2.1)	2 (1.1)	
In a relationship (without living together)	4 (2.1)	4 (2.3)	
Primiparous	140 (73.3)	122 (69.3)	0.71
Employment status			3.35
Employed	176 (92.1)	170 (96.6)	
Not currently working	15 (7.9)	6 (3.4)	
Educational level			5.66
Up to the 9th grade	2 (1.0)	4 (2.3)	
10th to 12th grade	30 (15.7)	26 (14.8)	
Bachelor’s degree	83 (43.5)	58 (33)	
Master’s or Doctorate	76 (39.8)	88 (50)	
Household monthly income			4.92
Less than 580€	8 (4.2)	9 (5.1)	
580–1000€	88 (46.1)	80 (45.5)	
1000–2000€	87 (45.5)	70 (39.8)	
More than 2000€	8 (4.2)	17 (9.7)	
Residence			1.06
Urban	141 (73.8)	138 (78.4)	
Rural	50 (26.2)	38 (21.6)	
Psychopathology history			6.86 *
Yes	48 (25.1)	25 (14.2)	
No	143 (74.9)	151 (85.8)	
Positive mental health			3.86 *
Flourishing	109 (57.1)	118 (67)	
Not flourishing	82 (42.9)	58 (33)	
Infant’s age (in months)	1.89 (0.94)	1.87 (1.32)	0.16
Infant’s sex			0.43
Male	98 (51.3)	93 (53.1)	
Infant’s gestational weeks (at birth)	38.89 (1.64)	38.95 (1.77)	−0.33

Note. * *p* < 0.05.

**Table 2:** Estimated marginal means and fixed effects for primary and secondary outcome measures.

Variables	Group	Time 1 M (SE)	Time 2 M (SE)	Effect	B (SE)	95% CI	*p*
**MHC-SF**	Intervention	51.17 (0.54)	53.94 (0.69)	Time	−2.77 (0.69)	[−4.13, −1.40]	<0.001
	Control	50.47 (0.57)	51.19 (0.61)	Group	−2.74 (0.93)	[−4.56, −0.92]	0.003
				Time × Group	2.05 (0.93)	[0.22, 3.87]	0.028
				Psychopathology history	0.43 (0.88)	[−1.31, 2.16]	0.630
				MHC-SF baseline category	13.75 (0.73)	[12.31, 15.18]	<0.001
				Infant’s age	−0.10 (0.31)	[−0.70, 0.50]	0.748
**EPDS**	Intervention	6.38 (0.26)	5.26 (0.33)	Time	1.12 (0.34)	[0.45, 1.80]	0.001
	Control	6.72 (0.27)	6.19 (0.29)	Group	0.93 (0.45)	[−0.06, 1.81]	0.036
				Time × Group	−0.60 (0.46)	[−1.50, 0.31]	0.194
				Psychopathology history	−1.43 (0.42)	[−2.25, −0.61]	0.001
				MHC-SF baseline category	−2.75 (0.34)	[−3.42, −2.08]	<0.001
				Infant’s age	−0.05 (0.14)	[−0.33, 0.23]	0.739
**HADS-A**	Intervention	4.40 (0.22)	3.88 (0.28)	Time	0.53 (0.29)	[−0.04, 1.09]	0.069
	Control	4.54 (0.23)	4.72 (0.25)	Group	0.84 (0.38)	[0.09, 1.59]	0.028
				Time × Group	−0.71 (0.38)	[−1.46, 0.05]	0.067
				Psychopathology history	−2.02 (0.36)	[−2.73, −1.31]	<0.001
				MHC-SF baseline category	−1.62 (0.30)	[−2.20, −1.04]	<0.001
				Infant’s age	0.05 (0.12)	[−0.20, 0.29]	0.710
**PMPS-E**	Intervention	69.06 (0.48)	73.09 (0.59)	Time	−4.11 (0.58)	[−5.14, −2.92]	<0.001
	Control	67.87 (0.50)	72.57 (0.53)	Group	−0.56 (0.81)	[−2.09, 1.05]	0.517
				Time × Group	−0.63 (0.77)	[−2.15, 0.81]	0.375
				Psychopathology history	−0.80 (0.79)	[−2.41, 0.72]	0.285
				MHC-SF baseline category	3.84 (0.65)	[2.54, 5.10]	<0.001
				Infant’s age	0.94 (0.27)	[0.40, 1.47]	0.001
**ES**	Intervention	61.05 (0.38)	61.52 (0.46)	Time	−0.47 (0.40)	[−1.27, 0.32]	0.244
	Control	61.32 (0.40)	61.05 (0.42)	Group	−0.47 (0.63)	[−1.70, 0.76]	0.454
				Time × Group	0.74 (0.54)	[−0.32, 1.79]	0.170
				Psychopathology history	0.73 (0.65)	[−0.55, 2.01]	0.264
				MHC-SF baseline category	4.29 (0.53)	[3.24, 5.34]	<0.001
				Infant’s age	−0.22 (0.22)	[−0.66, 0.22]	0.331
**IMS-S**	Intervention	6.54 (0.09)	6.36 (0.11)	Time	0.19 (0.09)	[0.00, 0.37]	0.046
	Control	6.48 (0.09)	6.30 (0.10)	Group	−0.05 (0.14)	[−0.33, 0.23]	0.718
				Time × Group	−0.02 (0.12)	[−0.26, 0.23]	0.901
				Psychopathology history	0.20 (0.15)	[−0.08, 0.49]	0.166
				MHC-SF baseline category	0.57 (0.12)	[0.33, 0.81]	<0.001
				Infant’s age	−0.11 (0.05)	[−0.21, −0.02]	0.024

Note. MHC-SF—Mental Health Continuum-Short Form; EPDS—Edinburgh Postnatal Depression Scale; HADS-A—Hospital Anxiety and Depression Scale-Anxiety; ES—Empowerment Scale; PMPSE - Perceived Maternal Parenting Self-Efficacy; IMS-S—Investment Model Scale-Satisfaction.
